# Process–Structure–Property Relationships in Boron-Doped CVD Diamond Films on Si_3_N_4_ for Biosensor Applications

**DOI:** 10.3390/ma19143027

**Published:** 2026-07-14

**Authors:** Susana Ferreira, André Costa Vieira, Miguel Neto

**Affiliations:** 1 Department of Mechanical Engineering and Industrial Management, Superior Technological School, Institute Polytechnic of Viseu, 3504-510 Viseu, Portugal; susana@estgv.ipv.pt; 2 CMAST—Centre for Mechanical and Aerospace Science and Technologies, University of Beira Interior, Rua Marquês de Ávila e Bolama, 6201-001 Covilhã, Portugal; 3 Department of Materials and Ceramic Engineering, CICECO, University of Aveiro, 3810-193 Aveiro, Portugal; mangelo@ua.pt

**Keywords:** boron-doped diamond, polycrystalline diamond, chemical vapor deposition (CVD), silicon nitride, biosensors

## Abstract

This study explores the direct growth of boron-doped diamond films on biocompatible silicon nitride (Si_3_N_4_) ceramic substrates using hot-filament chemical vapor deposition (HFCVD), with a view toward their use in implantable electrochemical biosensors. The focus of this work is the establishment of process–structure–property relationships relevant to biosensor performance, including microstructure, surface chemistry, wettability, and electrical behaviour. The effects of key deposition parameters, namely methane concentration, deposition pressure, and sample holder configuration, were analysed in relation to film microstructure, crystallographic orientation, surface chemistry, wettability, and electrical performance. Under low CH_4_/H_2_ ratios, microcrystalline diamond films with a pronounced (111) preferential orientation were obtained, enabling improved boron incorporation and electrical resistivity values within the range required for biosensor operation (≈1–10 kΩ). Surface analyses revealed partially hydrogen-terminated diamond layers enriched with oxygen-containing functional groups (C–O and C–O–C), which enhance surface wettability and are suitable for enzyme immobilization. Among the studied conditions, films deposited at 150 mbar and low methane flow displayed the most balanced combination of electrical conductivity, surface wettability, and microstructural stability. Overall, the results highlight the potential of boron-doped CVD diamond grown directly on Si_3_N_4_ as a robust and biocompatible material platform for future implantable biosensors, particularly for glucose monitoring applications.

## 1. Introduction

Diabetes mellitus remains one of the most significant global health challenges, with its prevalence continuing to increase worldwide. Current projections indicate that more than one-tenth of the global population may be affected in the coming decades [1,2,3]. Effective disease management relies on frequent monitoring of glucose levels, making reliable and accessible glucose detection technologies essential for both clinical practice and personalized healthcare [4,5]. Since the pioneering work of Clark and Lyons and the subsequent development of enzyme-based electrodes by Updike and Hicks, electrochemical methods have become the foundation of glucose monitoring technologies [6,7].

Beyond conventional blood analysis, glucose can also be monitored in alternative biological fluids such as saliva, sweat, urine, and interstitial fluid. However, the concentration range of glucose and the local physicochemical environment vary considerably among these media, creating additional requirements for sensor sensitivity, selectivity, and stability [8,9,10,11]. Among the different sensing approaches available, electrochemical biosensors remain the most widely implemented because they combine rapid response, high sensitivity, low manufacturing cost, and compatibility with miniaturized and portable devices [12,13,14]. Electrochemical biosensors operate through the integration of a biological recognition element with an electrochemical transducer, converting biochemical events into measurable electrical signals that can be used for quantitative analysis in medical, environmental, and biotechnological applications [15,16].

The performance of an electrochemical biosensor is strongly influenced by the properties of the electrode material. In this context, boron-doped diamond (BDD) has emerged as a particularly attractive platform because it combines excellent chemical stability, corrosion resistance, biocompatibility, low background currents, and a wide electrochemical potential window [17,18,19,20,21]. These characteristics enable reliable operation in complex biological environments while maintaining high analytical performance. BDD electrodes are commonly produced by chemical vapor deposition (CVD), a technique capable of generating conductive diamond coatings with controlled microstructures and doping levels [22,23,24]. Although CVD-grown diamond offers important advantages for sensing applications, the elevated deposition temperatures generally required during processing may limit substrate selection and integration strategies [25].

Considerable research efforts have therefore focused on exploiting diamond-based materials for sensing and biosensing applications [26,27,28,29,30,31,32]. In addition to their electrochemical properties, diamond surfaces can be chemically tailored through specific surface terminations and functionalization routes. Such modifications play a crucial role in controlling biomolecular interactions and facilitating the immobilization of enzymes, proteins, antibodies, and other biological recognition elements [33,34,35]. As a consequence, BDD-based systems have been investigated in a wide range of electrochemical sensing platforms, including enzymatic and non-enzymatic glucose sensors [36,37,38,39,40,41,42,43,44,45]. Furthermore, the combination of conductive diamond with metallic nanoparticles or catalytic surface modifications has been shown to enhance electron-transfer processes and improve sensing performance [42,43,44,45].

Another important advantage of BDD materials is their ability to maintain electrochemical activity under demanding operating conditions. Compared with conventional carbon electrodes, diamond-based electrodes exhibit superior chemical inertness, high resistance to oxidation, and excellent long-term stability in both aqueous and non-aqueous environments [46,47,48,49,50,51,52,53]. These attributes have contributed to the successful application of boron-doped nanocrystalline diamond electrodes for glucose determination in real biological samples, demonstrating high sensitivity, reproducibility, and operational stability [54]. Nevertheless, the practical implementation of diamond-based biosensors depends not only on the electrochemical behaviour of the active coating but also on achieving adequate electrical conductivity and robust electrical contact configurations.

Among potential substrate materials, silicon nitride (Si_3_N_4_) is particularly attractive because of its mechanical strength, chemical stability, wear resistance, and demonstrated biocompatibility [55,56,57]. Previous studies have confirmed the feasibility of depositing diamond coatings on Si_3_N_4_ ceramics; however, most investigations have primarily focused on coating adhesion, growth behaviour, or microstructural characterization [55,56,57]. Comparatively less attention has been devoted to understanding how deposition conditions affect the combination of structural, chemical, wetting, and electrical properties that ultimately govern biosensor performance.

The present work addresses this knowledge gap by investigating the direct growth of boron-doped microcrystalline and nanocrystalline diamond films on sintered Si_3_N_4_ substrates using hot-filament chemical vapor deposition (HFCVD) [55]. Particular attention is given to the establishment of process–structure–property relationships through a systematic evaluation of methane concentration, deposition pressure, and sample-holder configuration. The effects of these parameters on microstructure, crystallographic orientation, surface chemistry, wettability, and electrical resistivity are analysed in order to identify the conditions that are most suitable for future biosensor applications.

The novelty of this study lies in the integrated assessment of electrical performance and surface functionalization within the same processing framework. Specifically, the work demonstrates how deposition conditions can be tailored to achieve electrical resistivity values compatible with electrochemical sensing requirements while simultaneously promoting oxygen-containing surface functionalities that favour wettability and may support subsequent enzyme immobilization [17,18,19,33,34,35]. By establishing these correlations, this study provides design guidelines for the development of diamond–ceramic platforms intended for next-generation implantable electrochemical biosensors, with particular relevance to glucose-monitoring technologies.

## 2. Materials and Methods

### 2.1. Substrate Preparation and Diamond Film Deposition

**Figure 1:**
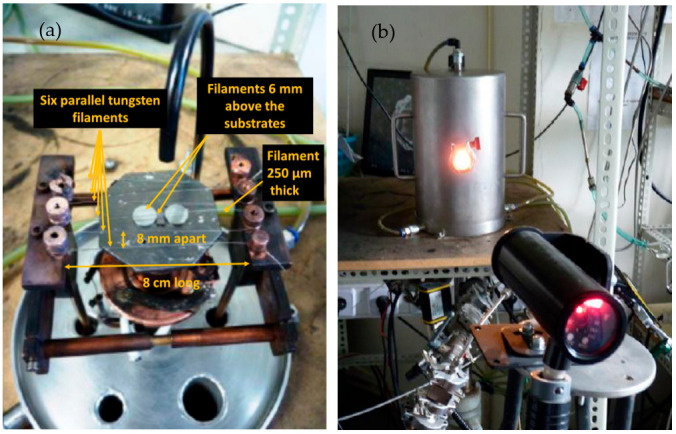
Deposition layout configuration: (**a**) Two Si_3_N_4_ ceramic disks placed inside the HFCVD chamber; (**b**) Complete HFCVD chamber assembled, showing the filament temperature measuring system, measuring through a chamber window.

For this study, boron-doped microcrystalline diamond (MCD) and boron-doped nanocrystalline diamond (NCD) were deposited on sintered silicon nitride (Si_3_N_4_) substrates measuring 10 mm in diameter and 1 mm in thickness. The discs were prepared from commercial ceramic powders according to an optimized process [58]. The surface preparation of these substrates involves subsequent polishing with 15 μm, 6 μm diamond, and colloidal silica (0.05 μm) to a mirror finish. Prior to diamond growth, the ceramic substrates were submitted to a diamond seeding process in which the mirror-polished ceramic surfaces were ultrasonically and mechanically abraded using a soft cloth (Gravimeta, Porto, Portugal) with 0.5–1 μm diamond powder (abcr GmbH, Karlsruhe, Germany) in an ethanol (Sigma-Aldrich, Burlington, MA, USA) suspension (0.1 g/mL). Using a home-built HFCVD equipment, boron-doped diamond layers were grown on the ceramic substrates for 3 h. Six parallel tungsten filaments (Goodfellow Advanced Materials, London, UK), 250 μm thick, 8 mm apart, 8 cm long, and placed 6 mm above the substrates, were used as heating elements in the HFCVD system (Figure 1).

The filament temperature was maintained between 2200 and 2300 °C, corresponding to a total power of approximately 1000 W. The hydrogen flow rate was kept constant at 128 sccm, while the methane flow was varied according to the experimental conditions defined in Table 1. Prior to deposition, a diamond seeding process was performed to increase nucleation density and ensure homogeneous film coverage. This step is critical for promoting film continuity and controlling grain size and microstructure. The doping source consisted of a boron oxide (B_2_O_3_) (abcr GmbH) solution dissolved in ethanol, with a B/C ratio of 5000 ppm. The precursor solution was prepared under controlled stirring conditions to ensure homogeneity and stability before deposition. It was subsequently introduced into the reaction chamber through a bubble system, using a controlled argon flow as a carrier gas. During deposition, the substrates were continuously rotated at a speed of 2 rpm. It should be noted that coating uniformity results from the combined effect of substrate rotation and the gas-flow-controlled deposition environment, rather than rotation alone. For each set of deposition parameters, three samples were produced to ensure reproducibility of the results. Two different sample holder configurations were investigated, namely Cu/Si_3_N_4_/Mo and Cu/Al_2_O_3_/Si_3_N_4_/Mo, in order to evaluate the influence of the thermal and chemical environment at the substrate interface. The corresponding deposition parameters are summarized in Table 1.

All experimental conditions were carefully controlled to ensure reproducibility and comparability between sample sets.

### 2.2. Characterization Techniques

For experimental characterization, different techniques were used. The BDD diamond surfaces were morphologically and microstructurally characterized by scanning electron microscopy (SEM, Hitachi S4100, Tokyo, Japan). To evaluate the surface roughness and the incorporation of sp^2^/sp^3^ carbon, atomic force microscopy (AFM, Nanoscope IIIa, Santa Barbara, CA, USA) analysis performed on a representative area of 50 µm × 50 µm) and Raman spectroscopy (Raman, HORIBA Jobin-Yvon HR800UV, Kyoto, Japan) using the excitation line of a 350 nm He-Cd laser) were used, respectively. Electrical resistivity was calculated by measuring surface resistance using the Van der Pauw method, by means of four independent copper wires bonded to the diamond film with silver glue. The electrical contacts were manually applied following the same procedure for all samples to ensure reproducibility and comparability of the measurements. Although minor variations in contact quality may occur due to the manual application of the conductive silver paste, this effect is considered minimal and does not compromise the overall trends observed in the electrical characterization. Film thickness was estimated using the spectral reflectance technique in the backscatter configuration, assuming a constant refractive index of 2.5 for all films [59]. X-ray diffraction spectroscopy (XRD) helped to identify the dominant crystallographic planes in the films. The characterization was performed through a Philips X’Pert MPD X-ray diffractometer (XRD) (Almelo, The Netherlands) using the Bragg–Brentano geometry and a CuKα radiation source (λ = 1.5406 Å, 40 kV, 50 mA). The equipment contains a PW1711 (proportional) detector that was varied the 2θ angle from 42.5° to 45° in 0.02° steps for 5 s with a fixed incidence angle of 2°. The average crystallite/grain size was evaluated using low-incident-beam angle X-ray diffraction (LIBAD) combined with the Debye–Scherrer equation. The wettability of the coatings was evaluated by measuring the water contact angle using the sessile drop method (Dataphysics, model OCA 20) (Filderstadt, Germany). These measurements were performed at room temperature (T = 25 ± 1 °C) using 2 μL of distilled and deionized water droplets deposited on the diamond surfaces. The final contact angles were calculated from the average of five consecutive measurements taken on different areas of each film, 30 s after contact with the diamond surface. The use of five measurements was adopted to ensure statistical reliability and account for surface heterogeneities of the coatings. The 30 s delay was selected to allow droplet stabilization and minimize transient spreading effects, in accordance with common practices reported in contact angle measurements for solid surfaces [60]. The analysis of the surface chemistry of the deposited films was performed using the X-ray Photoelectron Spectroscopy (XPS) technique, using a VSW XPS system with a Class 100 energy analyzer (Multi technic) (Manchester, UK) [61]. The survey spectra were performed in a fixed transmission mode of the analyzer with a pass energy of 44 eV, i.e., FAT 44, while the detailed spectra were performed in FAT 22 mode. The analysis was performed using a non-monochromatic Mg Kα line (photon energy of 1256.3 eV). For the calibration of the energy axis, Ag (110) samples and polycrystalline Au samples (previously washed by ion sputtering) were used. The energy was calibrated to the peak line position of the Ag 3d5/2 (binding energy of 368.22 eV) and Au 4f7/2 (binding energy of 83.96 eV) lines. To analyze the state of surface connections, a Fourier Transform Infrared Spectroscopy (FTIR) was used (PerkinElmer Spectrum BX with a Specac golden gate ATR) (Shelton, CT, USA), using a resolution of 4 cm^−1^ and an acquisition speed of 32 scans/minute.

## 3. Results

### 3.1. SEM Morphology

**Figure 2:**
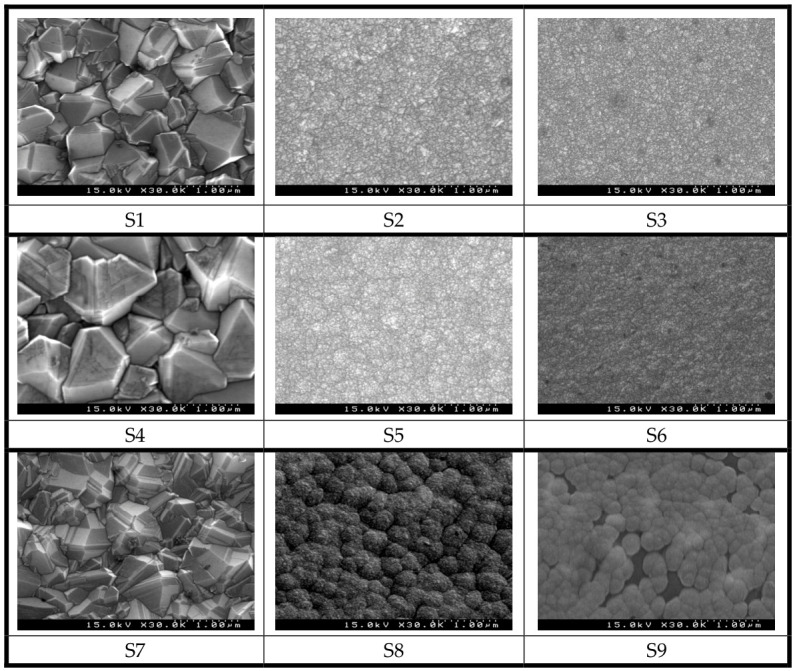
Top view SEM micrographs of the deposited diamond coatings. The scale bars in the images correspond to micrometer (µm) dimensions (S1–S9).

The SEM analysis of the deposited samples (Figure 2) shows that samples S1, S4, and S7 exhibit a well-defined faceted microstructure, typical of a microcrystalline diamond (MCD). Conversely, samples S2, S3, S5, and S6 are composed of much smaller grains of nanometer size, which is common of nanocrystalline diamond (NCD). Finally, samples S8 and S9 evidence a typical “ball-like” NCD microstructure, with an incomplete film for S9.

### 3.2. XRD and LIBAD

**Figure 3:**
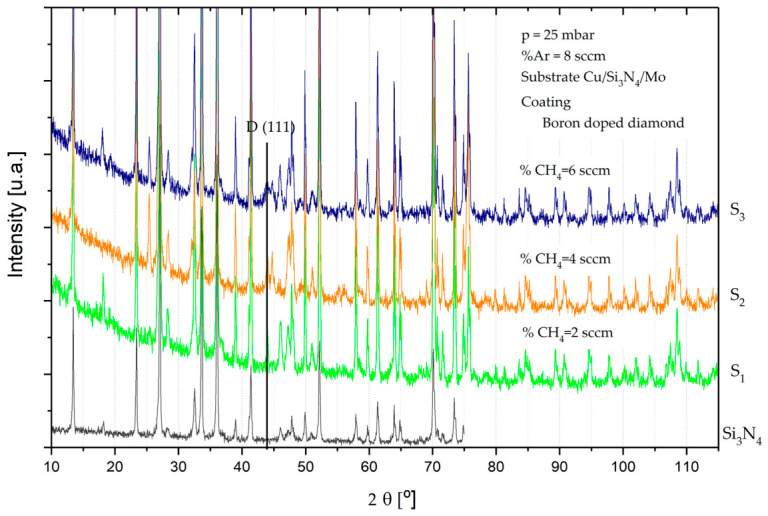
Conventional X-ray diffraction spectra of samples S1, S2 and S3.

**Figure 4:**
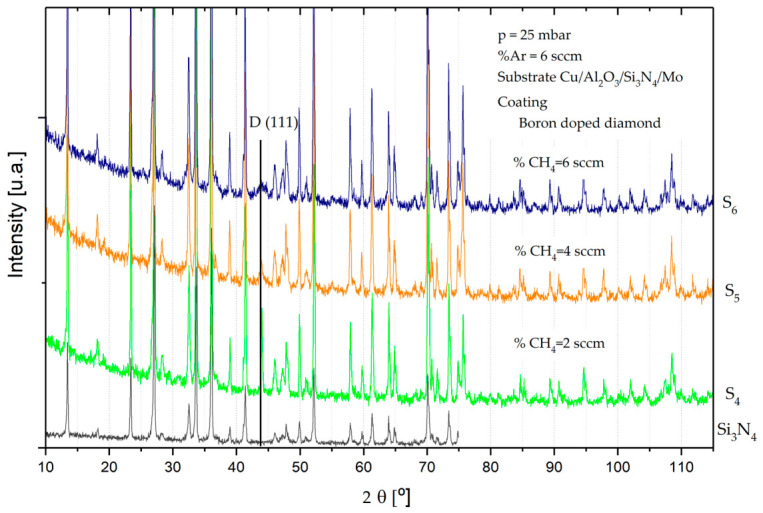
Conventional X-ray diffraction spectra of samples S4, S5 and S6.

**Figure 5:**
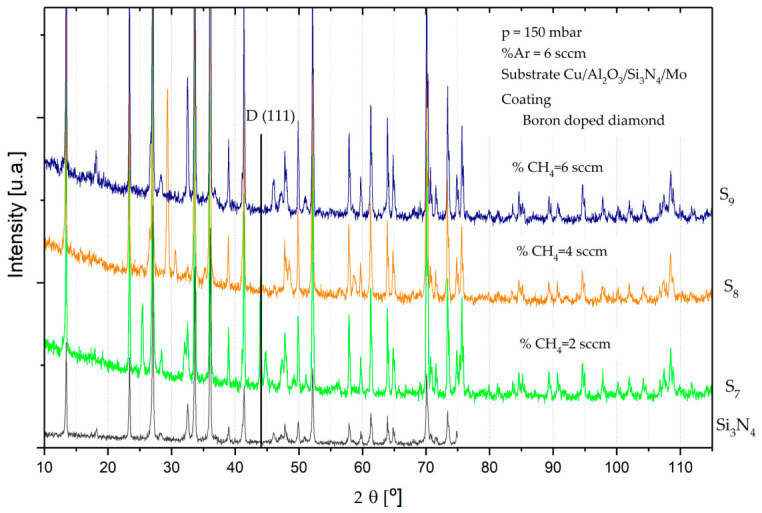
Conventional X-ray diffraction spectra of samples S7, S8 and S9.

The crystalline structure of the deposited films, using the XRD technique, is shown in the spectra of Figure 3, Figure 4 and Figure 5 (spectra obtained from the diffraction analysis performed in conventional mode).

**Figure 6:**
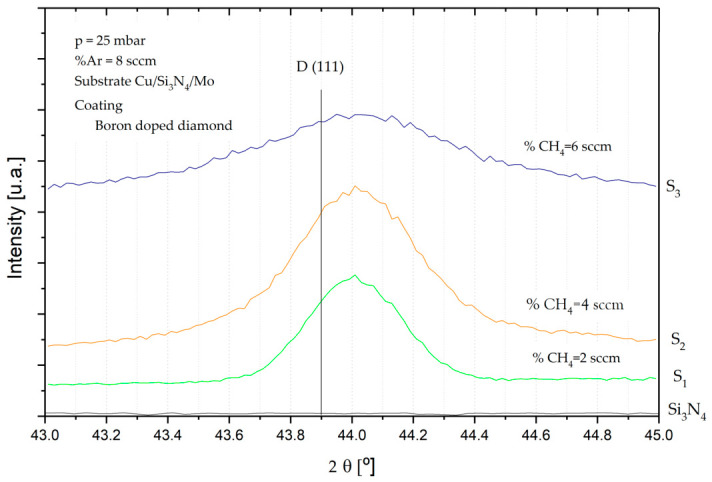
LIBAD spectra of samples S1, S2 and S3.

**Figure 7:**
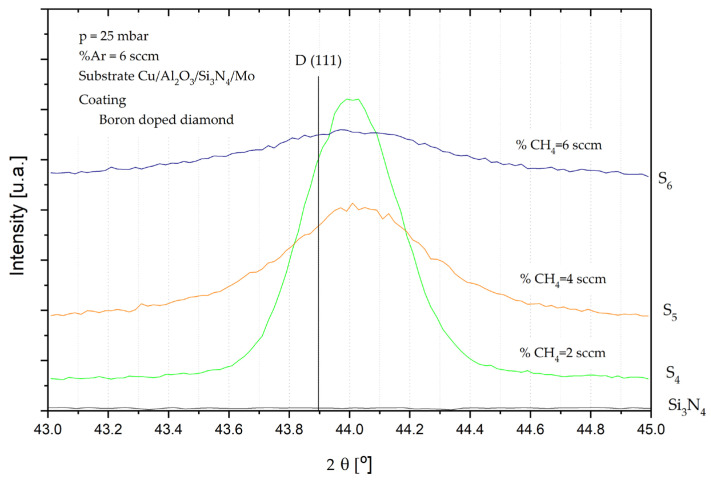
LIBAD spectra of samples S4, S5 and S6.

**Figure 8:**
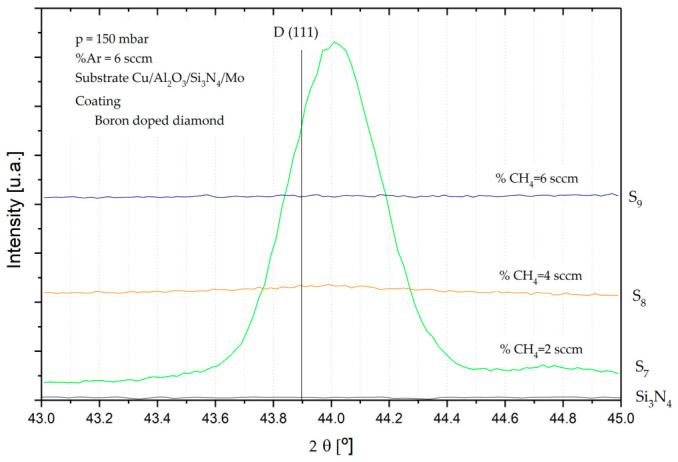
LIBAD spectra of samples S7, S8 and S9.

The results of XRD technique enabled to confirm that in addition to the peaks that are indexed to the β-Si_3_N_4_ substrate [62], it is possible to identify the presence of crystallographic (111) diamond planes in samples S1, S2, S3, S4, S5, S6 and S7, corresponding to a 2θ of 43.91°. To enable a more detailed analysis of the results, new diffraction tests were carried out in grazing mode to identify the presence of diamond (Figure 6, Figure 7 and Figure 8). The values of the average crystallite sizes obtained by Low Incident Beam Angle Diffraction (LIBAD) were: S1 (286 nm); S2 (54 nm); S3 (21 nm); S4 (214 nm); S5 (39 nm); S6 (23 nm); S7 (214 nm); S8 (32 nm); S9 (missing). The results show that samples S1, S4 and S7 have a micrometric crystallite size (MCD) while samples S2, S3, S5, S6 and S8 have a mean nanometric crystallite size (NDC).

### 3.3. Raman Spectroscopy

**Figure 9:**
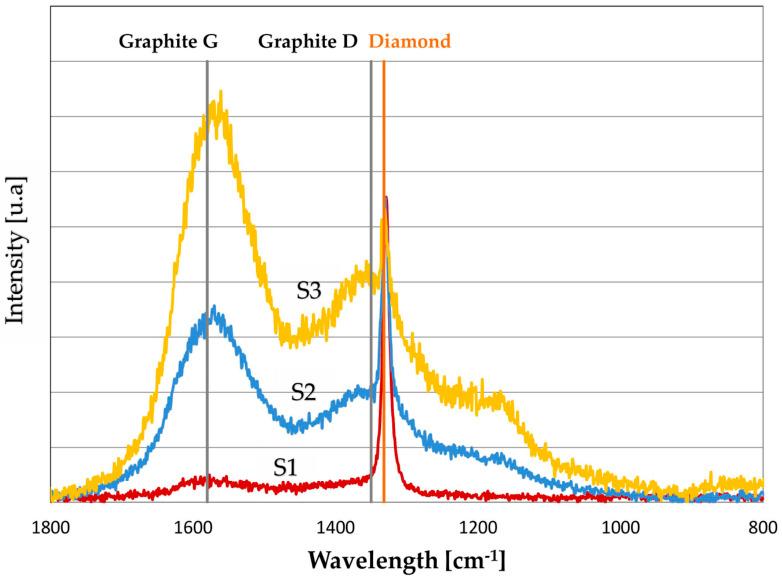
Raman spectra of samples S1, S2 and S3.

**Figure 10:**
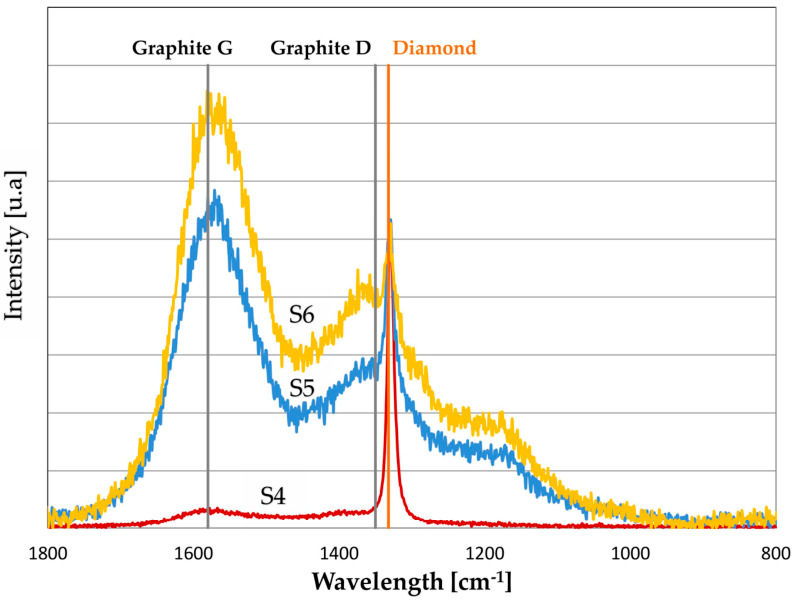
Raman spectra of samples S4, S5 and S6.

**Figure 11:**
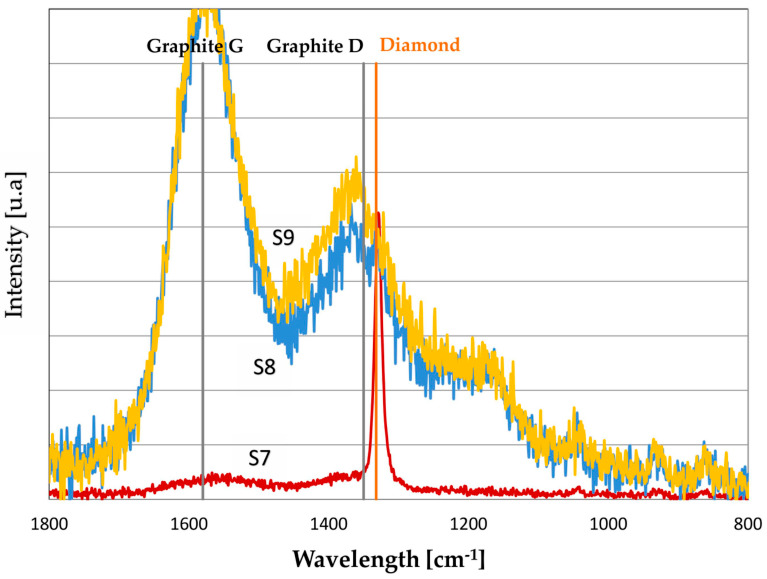
Raman spectra of samples S7, S8 and S9.

The results of the analysis of Raman spectra (Figure 9, Figure 10 and Figure 11) show that samples S1, S4, and S7 present the most intense diamond phases.

### 3.4. AFM Roughness

**Figure 12:**
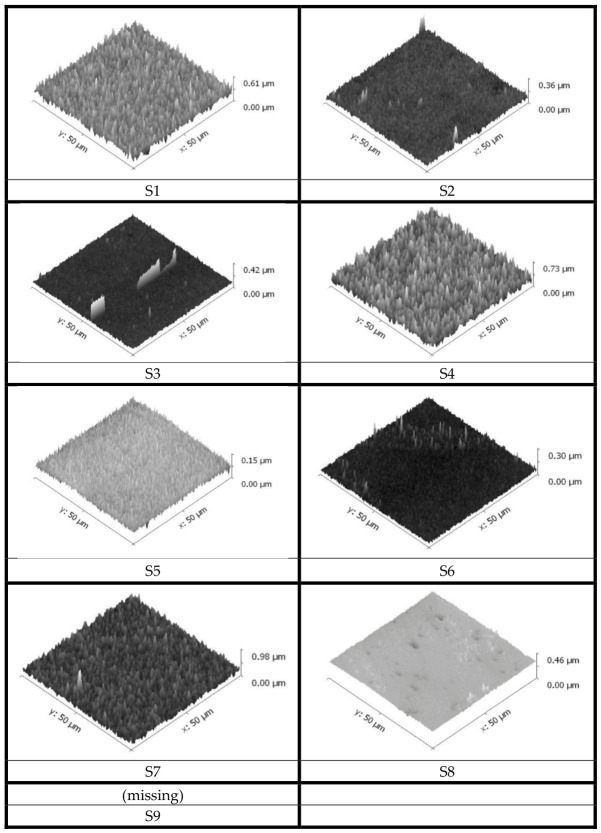
AFM images and corresponding surface roughness (Rq) values for samples (S1) to (S9).

Atomic force microscopy (AFM), presented in Figure 12, displays the surface roughness of the deposited coatings. The measured surface root-mean-squared roughness values obtained (R_q_) were: S1 (63.6 nm); S2 (21.5 nm); S3 (18.1 nm); S4 (90.9 nm); S5 (13.6 nm); S6 (13.7 nm); S7 (71.4 nm); S8 (15.7 nm); S9 (missing).

### 3.5. Wettability

**Figure 13:**
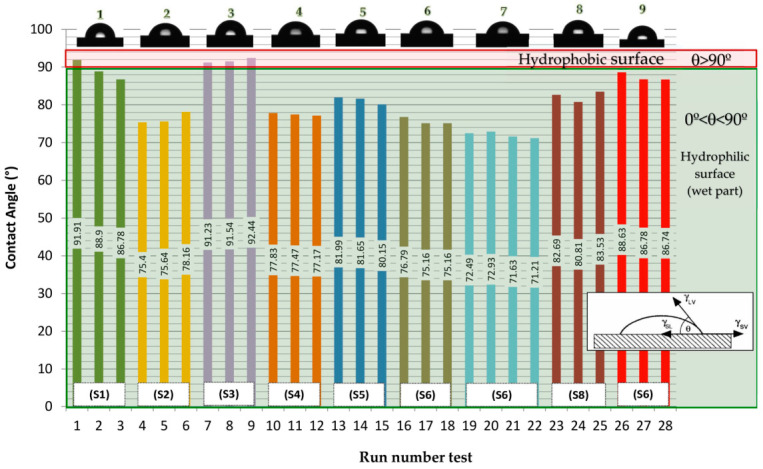
Contact angles (°) of a micro drop of water, measured 30 s after contact with the surface of samples S1 to S9.

The graph in Figure 13 shows the contact angles (°) of a micro drop of water with the surface of samples S1 to S9. The results of this analysis revealed that samples S1 and S3 exhibit hydrophobic surface behavior. It is also possible to observe that samples S5, S8, and S9 presented angle values very close to a hydrophobic surface, that is 81°, 83° and 87°, respectively (80–90° range). However, it is possible to note that samples S2, S4, S6 and S7 presented a hydrophilic surface behavior (partially wet).

### 3.6. FTIR

**Figure 14:**
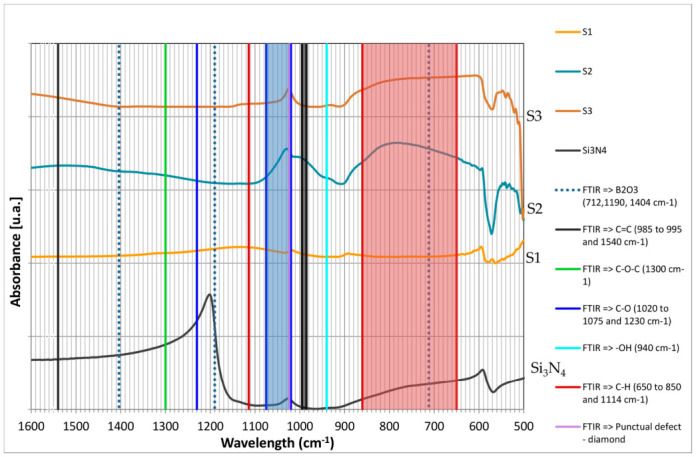
Absorption regions of the FTIR spectra of samples S1, S2 and S3.

**Figure 15:**
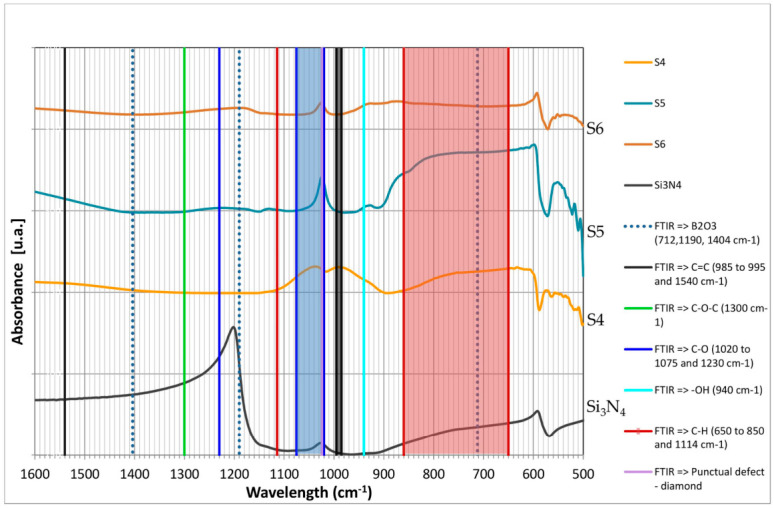
Absorption regions of the FTIR spectra of samples S4, S5 and S6.

**Figure 16:**
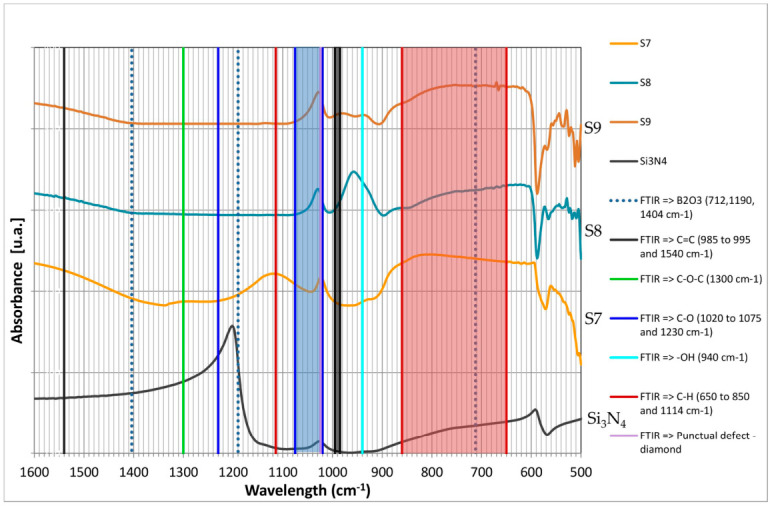
Absorption regions of the FTIR spectra of samples S7, S8 and S9.

**Figure 17:**
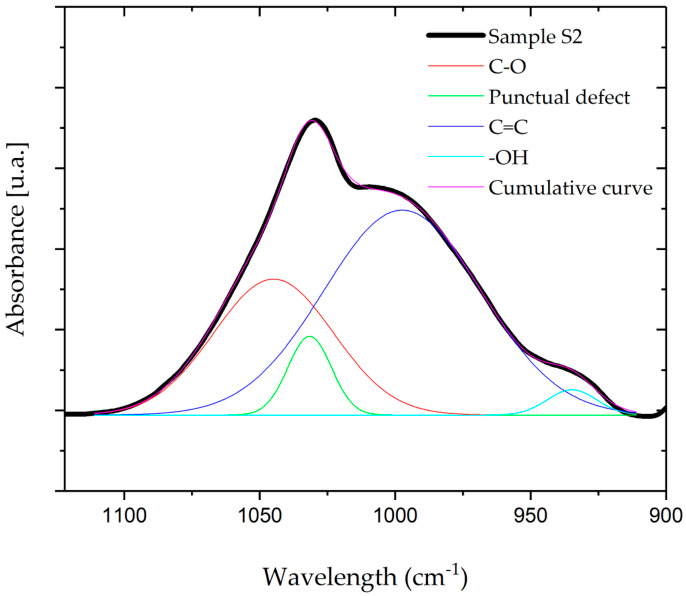
Deconvolution of the FTIR spectrum peaks of samples S2 (graph).

**Figure 18:**
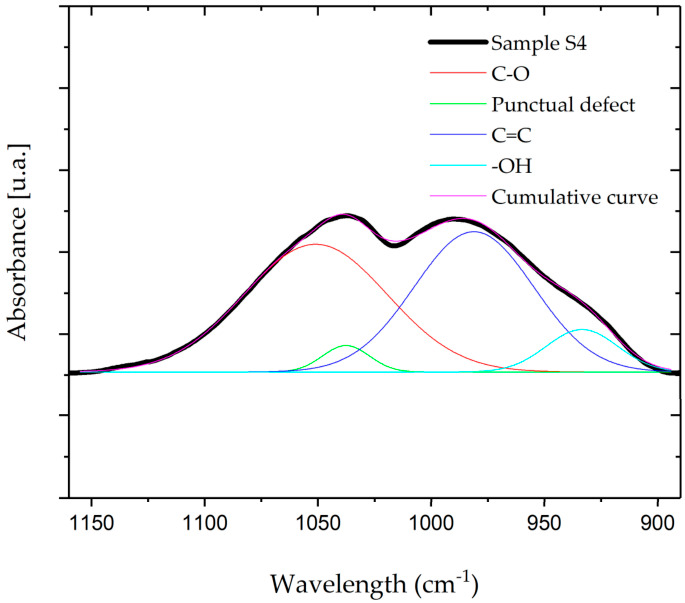
Deconvolution of the FTIR spectrum peaks of samples S4 (graph).

**Figure 19:**
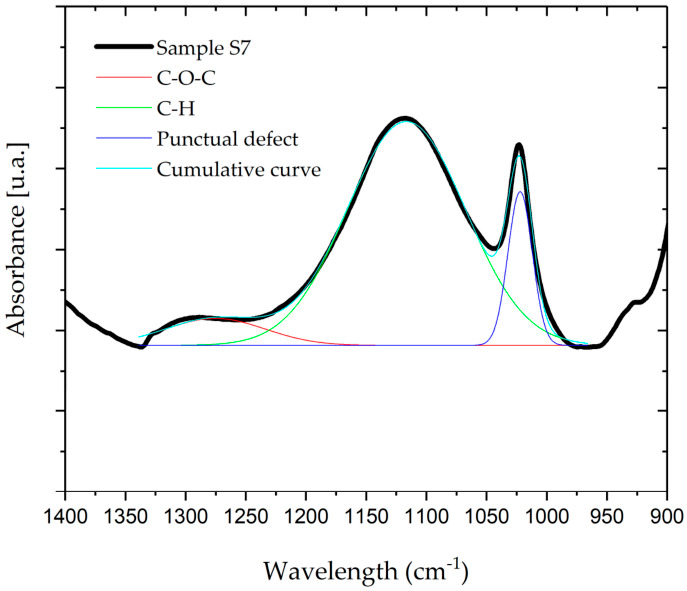
Deconvolution of the FTIR spectrum peaks of samples S7 (graph).

FTIR spectroscopy was used to identify surface functional groups and bonding configurations (Figure 14, Figure 15 and Figure 16). Table 2 summarizes the elongations of the groups in the respective absorption regions. Samples S2, S4, and S7 showed pronounced absorption bands associated with oxygen-containing functional groups. Deconvolution of the FTIR peaks (Figure 17, Figure 18 and Figure 19 and Table 3, Table 4 and Table 5) revealed that samples S2 and S4 are dominated by C–O bonds, while sample S7 exhibited stronger C–O–C contributions, along with –OH terminations.

### 3.7. XPS

X-ray photoelectron spectroscopy (XPS) analysis confirmed that the surfaces of all samples are primarily composed of carbon and oxygen, with minor contributions from silicon, originating from the substrate. Trace amounts of N, Na, S, and W were occasionally detected. Quantitative analysis (Table 6) showed that carbon is the dominant surface element in all coatings, followed by oxygen.

### 3.8. Electrical Properties

**Figure 20:**
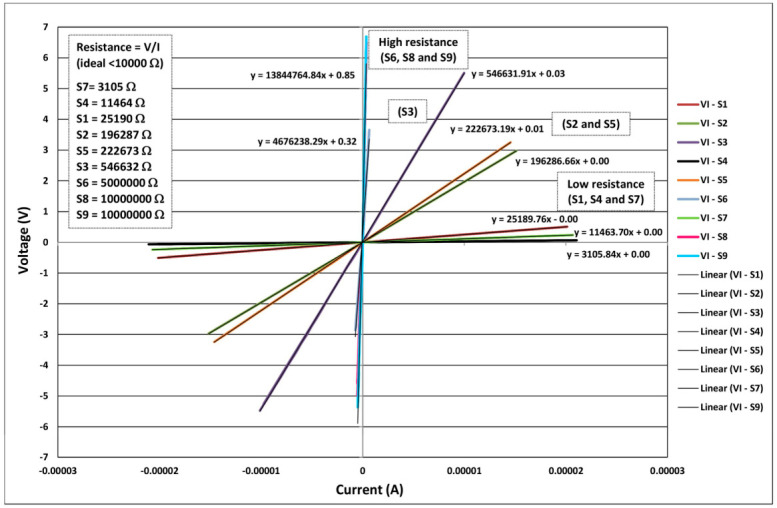
Electrical test results for samples S1 to S9.

As shown in Figure 20, samples S1, S4, and S7 exhibit the lowest resistance among all samples.

## 4. Discussion

The selected growth parameters studied in this work are based on knowledge acquired over the years, as they directly influence the characteristics of the diamond film. As an example, the work carried out by M. A. Neto [63] revealed the importance of argon flow in influencing the morphology of MDC coatings. The results of their work show that the surfaces of the deposited diamond are hydrophobic, that is, presumably hydrogen-terminated, with water contact angles of approximately 90°. However, it was found that these surfaces were only partially hydrogenated, revealing the presence of significant amounts of oxygen. Experimental data demonstrated that the presence of oxygen is not restricted to the surface but is also incorporated into the film during the CVD process. This situation was explained by specific gas chemistry, when gases resulting from ethanol, boron oxide, hydrogen, and methane come into contact with the hot tungsten filaments. Hydrogen reduction and carbothermal processes were also described as having a detrimental effect on the efficiency of boron doping in systems operating at higher pressures or using higher CH_4_/H_2_ flow ratios. Oxygen incorporation occurred through bonding with carbon atoms, mainly in the form of functional groups such as C–O–C and C=O. It is also important to highlight that two distinct groups could be identified based on the resulting microstructures. One group consists of well-defined microcrystalline grains with sizes ranging from 0.1 to 1 µm (MCD), while the other was composed of much smaller diamond grains (<100 nm), characteristic of a nanocrystalline diamond (NCD) morphology. Their results showed that MCD films were obtained for CH_4_/H_2_ ratios of 0.016 at a substrate temperature (T_s_) of 850 °C, whereas NCD films were formed at higher CH_4_/H_2_ ratios (0.032–0.064) and lower T_s_ (750 °C). Higher CH_4_/H_2_ ratios promoted diamond nucleation, while higher thermal energy favoured diamond grain growth. Furthermore, within each microstructure group, increasing the gas pressure, while keeping all other parameters constant, led to thinner MCD and NCD films. In the case of MCD, higher pressure also resulted in a slight reduction in grain size. The presence of argon during the boron-free diamond growth phase promoted secondary nucleation, which, either through increased gas pressure or argon flow, resulted in a decrease in crystallite size and growth rate.

Ultimately, important characteristics that may affect the film’s application as a biosensor included surface roughness, sp^2^ carbon content, boron doping level, surface functionalization, and wettability. It can be verified that the deposition process itself has a direct influence on the natural state of the surfaces after HFCVD diamond coating deposition.

The analysis of results obtained by comparing samples S1 and S3, S4 and S6, and S7 and S9 allows verification that reducing the CH_4_ flux in the deposition parameters contributes to obtaining films preferentially oriented along the (111) diamond plane. As the CH_4_ flow rate increases, the crystallinity of the diamond films decreases, accompanied by an increase in non-diamond carbon (sp^2^) phases. This behaviour was attributed to a reduced amount of atomic hydrogen in the reactor, which was responsible for the removal of non-diamond carbon species formed on the film surface. It is important to highlight that a lower presence of sp^2^ phases (graphite) contributes to greater biocompatibility of the deposited coating. A higher CH_4_/H_2_ ratio implies a greater amount of CH_x_ species in the gas phase and a lower concentration of atomic hydrogen. These observations are consistent with the Raman analysis, which revealed the coexistence of sp^3^ (diamond) and sp^2^ (graphitic) phases in the coatings. In particular, samples with higher sp^2^ carbon contributions tend to exhibit increased structural disorder, which can negatively affect charge transport. Conversely, films with more pronounced sp^3^ bonding and preferential (111) orientation show improved electrical performance, highlighting the direct correlation between phase composition and the measured resistivity.

Although comparisons between samples S4 and S7, S5 and S8, and S6 and S9 did not allow fully substantiated conclusions, it was nevertheless possible to infer that an increase in deposition pressure was an additional factor contributing to a reduction in diamond film crystallinity. This effect was associated with increased crystallographic disorder, as well as a broader dispersion in average crystallite size.

The obtained microstructures were closely linked to the respective deposition conditions, particularly to increases in the CH_4_/H_2_ ratio at constant pressure, which had favoured renucleation processes and consequently had reduced crystallite size [59,61,63]. Additionally, samples S8 and S9 showed that increasing the pressure by a factor of six (from 25 mbar to 150 mbar), combined with an increased CH_4_/H_2_ ratio, led to an undesirable morphology characterized by irregular surfaces and aggregates.

Samples S1, S4, and S7 exhibited the most pronounced preferential orientation along the (111) plane. According to the literature [63], this plane favours boron incorporation and minimizes dopant segregation at grain boundaries, allowing the films to exhibit higher electrical conductivity. Consequently, these samples displayed more effective boron incorporation, with a lower fraction of dopants located at grain boundaries, which is favourable for coating biocompatibility. On the other hand, these samples also presented the largest crystallite sizes (MCD). BDD coatings with smaller crystallite sizes provided a larger surface area for enzyme immobilization. However, very small crystallites may lead to increased enzyme loading and blocking effects due to enzyme–enzyme interactions. Therefore, an optimal crystallite size provides sufficient surface area for immobilization while preventing undesired interactions. In this context, microcrystalline BDD is particularly well suited for glucose biosensor applications.

Based on previous studies [63], characteristic absorption bands corresponding to C–O, C–O–C, C=O, and –OH groups were identified. Analysis of the spectral results as a function of absorption regions, together with wettability tests, confirmed that samples S2, S4, and S7, those exhibiting the highest surface wettability, also showed spectral peaks associated with bonds that promoted enhanced wettability (hydrophilic surfaces), namely C–O and C–O–C bonds, with pronounced absorption bands linked to oxygen-containing functional groups. These chemical features were directly associated with the observed wettability behaviour.

Given that this analysis aims to identify the factors that enable glucose testing in the environment where the sensor is deployed, a more detailed analysis was conducted by deconvoluting the spectral peaks of the aforementioned samples to identify specific surface bonds. Deconvolution of peaks for samples S2 and S4 revealed the presence of C–O bonds (hydrophilic surfaces) as well as –OH bonds. In contrast, sample S7 exhibited C–O–C bonds instead of C–O bonds, although –OH bonds were also present.

Surface analysis revealed that the main elements present were carbon, oxygen, and silicon. In some samples, residual amounts of nitrogen, sodium, sulfur, and tungsten were also detected which could be attributed to contamination of the deposition chamber from the tungsten filaments and cleaning products. Quantification of emitted photoelectrons enabled a compositional analysis confirming that carbon was the predominant element, followed by oxygen, with the remaining elements present at low atomic percentages. Oxygen presence was consistent with both FTIR results and previous reports indicating partial oxidation of diamond surfaces during HFCVD growth using boron oxide dissolved in ethanol precursors [64].

These observations are further supported by the underlying gas-phase chemistry occurring during the HFCVD process. Oxygen incorporation in the films can be attributed to the decomposition of ethanol and to plasma-induced reactions involving boron oxide precursors, which generate reactive oxygen-containing species in the deposition environment. These species interact with the growing diamond surface, leading to the formation of functional groups such as C–O and C–O–C.

From a functional perspective, the presence of these oxygen-containing groups plays a key role in modifying surface wettability and is particularly relevant for biosensor applications, as it facilitates subsequent enzyme immobilization. This aspect reinforces the potential of the developed coatings for electrochemical sensing platforms.

In addition, the trace amounts of tungsten detected by XPS are associated with filament evaporation during the HFCVD process. Although the detected levels are very low and are not expected to significantly affect the physicochemical properties of the films, this aspect is acknowledged as a potential limitation and will be addressed in future studies involving biological validation.

Therefore, controlling oxygen incorporation during deposition represents a key parameter for tailoring surface functionality toward specific biosensing applications.

Optimizing the electrical properties of a biosensor is crucial for its performance. Efficient operation requires the electrical resistance of the active material to be below 10 kΩ, preferably within the range of 1–10 kΩ (R = V/I < 10 kΩ). Electrical conductivity was evaluated by measuring potential as a function of current. The results indicated that samples S1, S4, and S7 exhibited the lowest electrical resistance. However, only sample S7 fulfilled the ideal conditions for electrical conductivity, while sample S4, although outside optimal conditions, presented values close to those required for biosensor applications. These findings support the conclusion that microcrystalline films were more favourable for boron doping, as they exhibit preferential (111) orientation, which enhances conductivity due to increased boron incorporation at grain boundaries.

## 5. Outlook and Limitations

Although the present study provides a comprehensive analysis of the structural, morphological, surface chemical, and electrical properties of boron-doped diamond coatings deposited on Si_3_N_4_ substrates, some limitations should be acknowledged.

First, the scope of this work is restricted to physicochemical characterization. No biological validation, such as cytotoxicity, hemocompatibility, or long-term stability in physiological environments, was performed. These aspects are critical for confirming the suitability of the developed coatings for implantable biosensor applications and will be addressed in future studies.

Second, the study focuses on material-level performance, and therefore does not include direct demonstration of biosensing functionality, such as enzyme immobilization efficiency, electrochemical sensitivity, or glucose detection capability in real media. Future work will involve the integration of biomolecular recognition elements and the evaluation of device-level performance.

Another limitation concerns the experimental design, in which multiple parameters (e.g., substrate holder configuration and gas-phase composition) may simultaneously influence the observed results. Although trends have been identified, further studies under more controlled conditions will be necessary to fully decouple these effects and refine process optimization strategies.

In addition, trace contamination from tungsten filaments was detected, although at very low levels. While not expected to significantly affect material performance, its potential impact on biological response remains to be clarified.

From a technological perspective, the current study is limited to a laboratory-scale HFCVD system. Therefore, issues related to scalability, process reproducibility at industrial level, and economic feasibility still need to be evaluated before practical implementation.

Despite these limitations, the results presented here establish a solid foundation for future development of diamond-based biosensor platforms. In particular, the ability to tailor surface chemistry and electrical properties through process control represents a promising route for the design of next-generation implantable electrochemical sensors.

Further optimization of process parameters will be essential to achieve reproducible and application-specific performance.

## 6. Conclusions

The present work demonstrates that β-Si3N4 substrates are suitable for the direct growth of boron-doped diamond films with surface resistivities compatible with biosensing applications, confirming their potential as a robust platform for electrochemical devices. The obtained results clearly highlight the critical role of deposition parameters, particularly CH_4_ flow rate and system pressure, in determining the microstructure, surface chemistry, wettability, and electrical performance of the coatings. In particular, a deposition pressure of 150 mbar combined with a low CH_4_ flow rate (2 sccm) enables the formation of microcrystalline diamond films with properties most suitable for biosensor applications.

An increase in deposition pressure was found to promote the formation of oxygen-containing functional groups, such as C–O–C bonds, which contribute to enhanced surface wettability. In contrast, lower pressures tend to favour the formation of C–O bonds, although both configurations exhibit hydrophilic behaviour. These surface functionalities play a key role in enabling interaction with biological species, which is essential for biosensor applications. Furthermore, lower CH_4_ flow rates were shown to favour the development of surface chemistries with enhanced hydrophilic characteristics, which are particularly advantageous for enzyme immobilization processes.

Despite the predominance of hydrophilic behaviour, all samples exhibited a coexistence of hydrophilic and hydrophobic terminations, indicating that the diamond surfaces are only partially hydrogenated. This effect is attributed to the hydrogen-rich atmosphere present during the deposition process, which promotes surface hydrogen termination. At the same time, oxygen incorporation was observed not only at the surface but also within the film, resulting from the complex gas-phase chemistry involving ethanol, boron precursors, and methane in the presence of hot tungsten filaments.

Overall, the results establish a clear relationship between deposition conditions and the resulting material properties, demonstrating that controlled HFCVD processing enables the tailoring of boron-doped diamond coatings with characteristics suitable for future implantable electrochemical biosensors, particularly in applications requiring a balance between electrical conductivity and surface functionalization.

## Figures and Tables

**Table 1:** Deposition parameters.

Samples	Deposition Pressure [mbar]	CH_4_ [sccm ^1^]	H_2_ [sccm ^1^]	Ar [sccm ^1^]	Sample Support
S1	25	2	128	8	Cu/Si_3_N_4_/Mo
S2	25	4	128	8	Cu/Si_3_N_4_/Mo
S3	25	6	128	8	Cu/Si_3_N_4_/Mo
S4	25	2	128	6	Cu/Al_2_O_3_/Si_3_N_4_/Mo
S5	25	4	128	6	Cu/Al_2_O_3_/Si_3_N_4_/Mo
S6	25	6	128	6	Cu/Al_2_O_3_/Si_3_N_4_/Mo
S7	150	2	128	6	Cu/Al_2_O_3_/Si_3_N_4_/Mo
S8	150	4	128	6	Cu/Al_2_O_3_/Si_3_N_4_/Mo
S9	150	6	128	6	Cu/Al_2_O_3_/Si_3_N_4_/Mo

^1^ sccm—Standard Cubic Centimeters per Minute.

**Table 2:** Elongations of the groups in their respective absorption regions (FTIR).

Absorption Regions Vibration Frequencies [cm^−1^]	Group Elongation Vibrations	Observations
2500–3600	–OH	More hydrophobic surfaces
2000–2300	C connections ≡W	Possibility of acetylene (C2H2) being formed and incorporated during the CVD process in the grain boundaries of the samples in question
≈1920	pairs of the type C=X=Y	Since boron and oxygen are elements present during the CVD process, they can be incorporated into these vibrations as X or Y.
≈1700	C=O bonds	Example of ketone, ester or carboxylic acid
≈1540	C=C bonds	It is associated with a greater crystallinity of the diamond film
≈1300	C–O–C bonds	Surfaces with greater wettability
≈1230	C–O bonds	Surfaces with greater wettability
≈1024	punctual defect	Vibration does not come from the surface, but from a point defect characteristic of the samples in the diamond’s crystal lattice
≈940	Bending vibrations –OH	For example, from a carboxylic acid. These vibrations, if they exist, must be originated from –OH groups present at the grain boundaries of the diamond films and not at the surface.
650–860	C–H bending vibrations	It may be related to hydrogen bonding to carbon

**Table 3:** Deconvolution of the FTIR spectrum peaks of samples S2 (table).

Model	Gaussian			
Equation	y = y0 + A/(w × sqrt(pi/(4 × ln(2)))) × exp(−4 × ln(2) × (x − xc)^2^/w^2^)			
Plot	Peak1(B)	Peak2(B)	Peak3(B)	Peak4(B)
y0	100.57721 ± 0.07869	100.57721 ± 0.07869	100.57721 ± 0.07869	100.57721 ± 0.07869
xc	1044.85602 ± 1.62759	1031.55584 ± 0.11134	997.38514 ± 1.56412	934.72477 ± 0.31964
A	−929.16992 ± 110.30376	−196.06195 ± 12.80877	−1845.10333 ± 98.75384	−71.02421 ± 6.25379
w	51.83325 ± 1.57332	18.87732 ± 0.50548	68.26902 ± 1.97742	21.33744 ± 1.13059
Reduced Chi-Sqr	0.06808			
R-Square(COD)	0.99952			
Adj. R-Square	0.99949			

**Table 4:** Deconvolution of the FTIR spectrum peaks of samples S4 (table).

Model	Gaussian			
Equation	y = y0 + A/(w × sqrt(pi/(4 × ln(2)))) × exp(−4 × ln(2) × (x − xc)^2^/w^2^)			
Plot	Peak1(B)	Peak2(B)	Peak3(B)	Peak4(B)
y0	94.62357 ± 0.05808	94.62357 ± 0.05808	94.62357 ± 0.05808	94.62357 ± 0.05808
xc	1050.9698 ± 1.96084	1037.56734 ± 0.35191	981.08643 ± 0.80494	933.3181 ± 0.72821
A	−1235.64016 ± 78.50638	−83.70448 ± 16.10157	−1132.19589 ± 77.53529	−204.65177 ± 25.60769
w	74.52238 ± 2.23413	24.36935 ± 1.76417	62.16484 ± 2.85107	37.11192 ± 1.4937
Reduced Chi-Sqr	0.05434			
R-Square(COD)	0.9989			
Adj. R-Square	0.99885			

**Table 5:** Deconvolution of the FTIR spectrum peaks of samples S7 (table).

Model	Gaussian		
Equation	y = y0 + A/(w × sqrt(pi/(4 × ln(2)))) × exp(−4 × ln(2) × (x − xc)^2^/w^2^)		
Plot	Peak1(B)	Peak2(B)	Peak3(B)
y0	91.82791 ± 0.11554	91.82791 ± 0.11554	91.82791 ± 0.11554
xc	1275.30137 ± 1.47256	1116.61257 ± 0.18571	1022.12113 ± 0.10433
A	−346.18567 ± 20.74539	−3403.20529 ± 27.63495	−481.2175 ± 6.12273
w	97.75017 ± 4.40388	115.72309 ± 0.69807	23.8312 ± 0.27491
Reduced Chi-Sqr	0.31065		
R-Square(COD)	0.99643		
Adj. R-Square	0.99634		

**Table 6:** Atomic percentages of the chemical elements present in each deposited sample (XPS).

	Elements			
Samples	C 1 s	O 1 s	Si 2p	Others
S1	91.16	7.88	0.97	S
S2	91.92	7.69	0.39	N, Na and S
S3	92.47	7.39	0.14	S and W
S4	90.32	7.53	2.15	S
S5	93.36	6.57	0.07	S
S6	93.16	6.5	0.35	S
S7	92.99	6.93	0.09	N and S
S8	89.42	9.55	1.03	N and S
S9	90.58	9.01	0.41	S

## Data Availability

The original contributions presented in this study are included in the article. Further inquiries can be directed to the corresponding author.

## Abbreviations

The following abbreviations are used in this manuscript:

AFM	Atomic Force Microscopy
ATR	Attenuated Total Reflectance
BDD	Boron-Doped Diamond
BDND	Boron-Doped Nanocrystalline Diamond
B/C	Boron-to-Carbon ratio
CVD	Chemical Vapor Deposition
FTIR	Fourier Transform Infrared Spectroscopy
HFCVD	Hot Filament Chemical Vapor Deposition
IDF	International Diabetes Federation
LIBAD	Low Incident Beam Angle Diffraction
MCD	Microcrystalline Diamond
NCD	Nanocrystalline Diamond
Rms	Root Mean Square roughness
Sa	Arithmetic mean surface roughness
sccm	Standard Cubic Centimeters per Minute
SEM	Scanning Electron Microscopy
S/N	Signal-to-Noise ratio
T_s_	Substrate temperature
XPS	X-ray Photoelectron Spectroscopy
XRD	X-ray Diffraction
